# Abasic RNA: its formation and potential role in cellular stress response

**DOI:** 10.1080/15476286.2023.2223466

**Published:** 2023-06-15

**Authors:** Tanya Prashar, Fernand De La Selle, Katalin A. Hudak

**Affiliations:** Department of Biology, York University, Toronto, Canada

**Keywords:** Abasic RNA, RNA N-glycosylase, no-go decay, ribosome stalling, R-loops, ribotoxic stress response

## Abstract

RNA is integral to gene expression as messenger RNA (mRNA), transfer RNA (tRNA) and ribosomal RNA (rRNA) each play roles to transmit information from DNA into synthesis of functional proteins. During their lifespan, these nucleic acids may be chemically modified by alkylation, oxidation and the removal of bases, which alters their activity. Though much research has been devoted to the detection and repair of damaged DNA, RNA is viewed as a short-lived molecule that is quickly degraded upon damage. However, recent studies indicate that RNAs that become modified, particularly during stress, function as important signalling molecules. In this review, we focus on the effects of abasic RNAs and the modifications that lead to the loss of a base, as RNAs that are initially methylated or oxidized often become abasic. We describe how these chemical changes occur and cite recent work showing that in addition to being indicators of damage, abasic RNAs function as signals that mediate downstream cellular responses to stress.

## Introduction

Various chemical agents, ranging from environmental pollutants to metabolites from endogenous sources, may alter the composition of nucleic acids, thereby affecting their functions. Due to the reactivity of their oxygen and nitrogen atoms [1], nucleic acids are commonly modified by oxidation, alkylation and base removal. Apurinic/apyrimidinic (AP or abasic) sites refer to nucleotides with missing bases in DNA or RNA, formed by cleavage of the glycosidic bond joining the (deoxy)ribose sugar and the nitrogenous base. These modifications occur commonly in DNA and their repair follows well studied and conserved pathways to mitigate the effect of these lesions [2]. Comparatively less is understood about the nature and effects of abasic sites in RNA.

RNA is challenging to study because of its relatively transient nature, and its multiple copies suggest that any damage to individual molecules would not likely be permanently detrimental to the cell. Still, there are important biological consequences of abasic lesions in RNA. RNA is more abundant than DNA [3] and serves both coding and non-coding functions that encompass transcriptional and translational control of gene expression. Therefore, damage to RNA by the loss of bases impacts the cell in many ways. Several factors contribute to the propensity of RNA for base loss compared to DNA. Approximately 30–40% of RNA is single stranded and not protected by classic Watson-Crick base pairing and, unlike DNA, RNA is not closely associated with protective histones [1,4]. In addition, the RNA of eukaryotic cells is not sequestered into the nucleus but rather is exposed to increased numbers of chemical agents in the cytoplasm [5,6]. Initial *in vitro* studies characterized the resistance to depurination of RNA compared to DNA. Spontaneous generation of abasic RNA is slower than DNA due to the electron-withdrawing nature of the 2’-OH group of ribose which lowers the rate of hydrolysis [7,8]. However once formed, abasic RNA is several-fold more resistant to cleavage of the ribose-phosphate backbone than abasic DNA [9]. The increased stability of the resulting abasic RNA suggests downstream biological impact due to its greater longevity.

In this review, we describe how abasic sites form in mRNA, tRNA and rRNA and we examine their effects in cells. In addition to being damaged RNAs that must be detected and degraded, abasic RNAs are also important signalling molecules that communicate cell status and control gene expression.

## How abasic sites are formed

Abasic sites are formed in RNA by the hydrolysis of the glycosidic bond connecting the nitrogenous base to the ribose sugar. The process begins with the protonation of a nitrogen atom leaving a positive charge on the base. Usually, this occurs on purines rather than pyrimidines as the pyrimidine’s glycosyl bonds are 20-fold more stable than purinic bonds [10]. Purines can delocalize charge around their dual-ring system thereby promoting the displacement of the electrons forming the glycosidic bond [11], eventually resulting in cleavage. The loss of purine bases can also be due to hydrolysis from base methylation [12], and activity of free radicals [13] and RNA N-glycosylase enzymes [14]. We briefly review some of these mechanisms of formation below.

### Spontaneous hydrolysis

**Figure 1.**
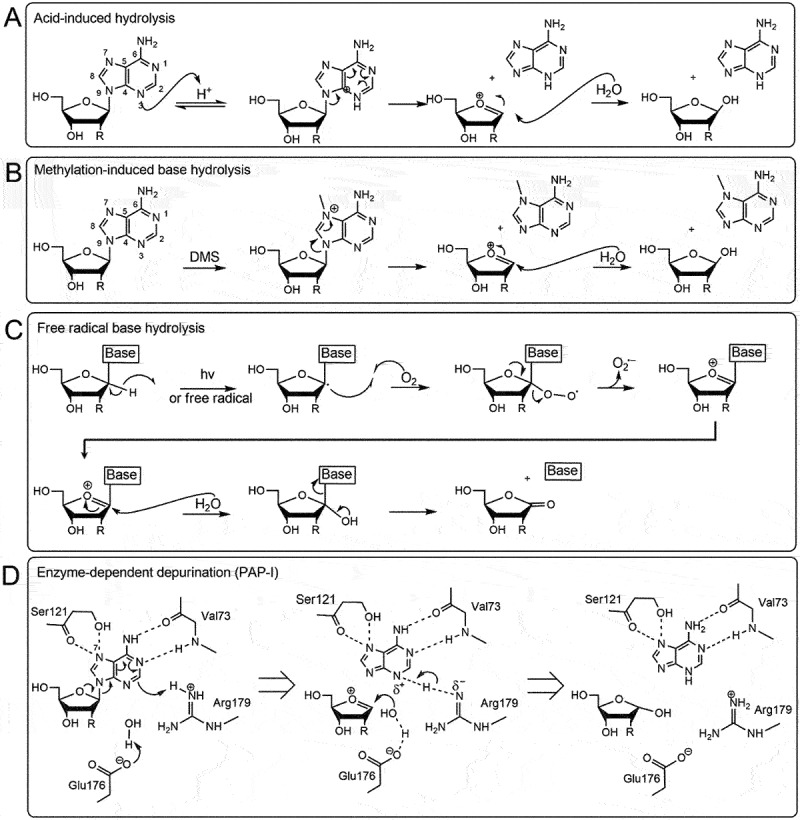
Chemical mechanisms of base excision from nucleic acids. A) Acid-induced hydrolysis relies on protons or hydrogen ions to protonate the adenine base which will trigger an electron delocalization around the dual ring eventually breaking the glycosidic bond. A nearby water molecule can resolve the oxocarbenium transition state. B) Methylation-induced base hydrolysis can occur when a base is methylated (for example by dimethyl sulfate, DMS) which will trigger an electron delocalization event severing the glycosidic bond. Like the acid-induced mechanism, a water molecule will resolve the transition state. C) in free radical base hydrolysis, the free electron displaces the hydrogen atom at the C1’ thereby producing superoxide. The oxocarbenium ion is resolved by a water molecule resulting in base excision and production of a carbonyl group on the C1’. D) an RNA N-glycosylase (pokeweed antiviral protein, PAP-I) removes the adenine by protonating the N−7 of the adenine with arginine 179. This will trigger an electron delocalization event thereby excising the glycosidic bond and producing an oxocarbenium ion that will be resolved by a water molecule directed by glutamic acid 176. Additional amino acids valine 73 and serine 121 help maintain the adenine in the correct orientation.

In a typical depurination setting enhanced by acidic conditions, the N-glycosidic bond is under threat by the protonation of the N−7 of purines. This protonation will create a rearrangement of electrons starting with the neighbouring double bond that will alleviate the localized positive charge on N−7 (Figure 1a). The charge delocalization continues with the formation of a double bond between N−9 and C−8 which occurs from the cleavage of the N-glycosidic bond and the formation of an oxocarbenium ion on the pentose sugar [11,15]. The hydrolysis reaction comes to completion when the charge on the sugar is resolved in the presence of a water molecule.

### Hydrolysis from base methylation

This mechanism is very similar to spontaneous hydrolysis described above in that a positive charge is induced on the nitrogenous ring [16]. The N−7 from the purine acquires a methyl either from alkylating agents such as dimethyl sulphate or from the activity of methyltransferases. Addition of the methyl group creates a localized positive charge which is alleviated by an oxocarbenium intermediate state followed by resolution from the water nucleophilic attack (Figure 1b).

### Hydrolysis from free radicals/oxidation

Reactive oxygen species (ROS) are commonly produced by normal aerobic metabolic processes and can be increased by environmental stressors such as ionizing radiation and pathogen attack [17]. They are derived from molecular oxygen and include superoxide anion radical (O_2_^•-^), hydrogen peroxide (H_2_O_2_) and hydroxyl radical (OH^−^). The mechanism of RNA depurination begins with the sequestration of the C1’ hydrogen atom from the ribose sugar by a superoxide radical which yields a peroxyl radical (Figure 1c). The superoxide radical is then ejected from the ribose forming a carbocation which becomes the target of the nucleophilic reaction with a water molecule. This reaction releases the base from the ribose to give the C1’ alcohol intermediate and the hydrolysis reaction ends with the formation of a ribonolactone from the hydroxyl that stabilizes into a carbonyl group [13].

### Hydrolysis from RNA N-glycosylase enzymes

RNA abasic sites can also be generated by RNA N-glycosylases (EC 3.2.2.22). These enzymes are produced in many plants, fungi and bacteria where they are described as toxins involved in pathogen defence [18]. These RNA N-glycosylases act primarily on the α-sarcin/ricin loop (SRL) of the 28S rRNA by removing an adenine (A2660 *E. coli*, A3027 yeast) within a GAGA tetraloop on the SRL. The conserved amino acids within the active site of glycosylases include a glutamic acid and an arginine which are the principal actors in depurination while amino acids valine and serine help to stabilize the adenine base [19]. Similar to previous depurination mechanisms, a positive charge arises from the protonation of N−3 by arginine, which in turn creates a cascade of electron rearrangement within the base (Figure 1d). Cleavage of the glycosidic bond between the ribose sugar and the nitrogenous base leaves an oxocarbenium that is resolved by the nucleophilic attack of a hydroxide molecule that was deprotonated by the glutamic acid [20,21].

**Figure 2.**
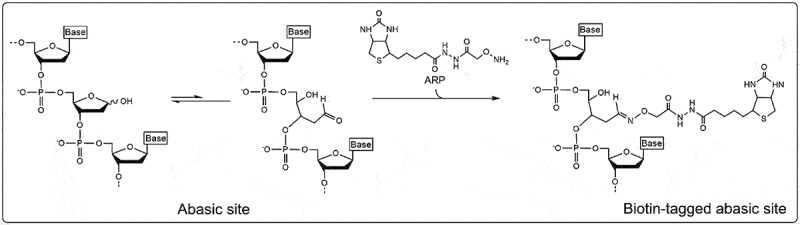
Reaction mechanism of aldehyde reactive probe (ARP). The abasic ribose exists in an unequal equilibrium between two conformations: the “closed” ring structure of the ribose and the “open” structure revealing an accessible aldehyde group. The aldehyde reactive probe is an amine, often biotinylated, that covalently binds with the exposed aldehyde group at the C1’ position of the abasic site.

Abasic sites in RNA from biological samples are often detected using an aldehyde reactive probe. The ribose lacking a base forms an unequal equilibrium between two conformations: the ‘closed’ ring structure of the ribose and the ‘open’ structure revealing an accessible aldehyde group (Figure 2). An aldehyde reactive probe, such as N′-aminoxymethylcarboxylhydrazino-D-biotin, is a biotinylated amine that covalently binds with the exposed aldehyde group at the C1’ position of the abasic site. These aldehyde groups form a Schiff base with the amino residues of the aldehyde. The addition of biotin to the probe allows for convenient detection of the abasic site by a number of streptavidin-based assays [22,23].

## Abasic mRNA

### Abasic mRNAs as indicators of cell stress

Abasic sites on mRNAs arise largely from hydrolysis of oxidized bases, which are formed from reaction with reactive oxygen species during normal cellular metabolism. For example, superoxide anion is produced by reaction of molecular oxygen with the reduced form of nicotinamide adenine dinucleotide (NADH), a component of the electron transport chain in mitochondria [24]. The superoxide anion in turn can produce other reactive oxygen species such as hydrogen peroxide and hydroxyl radical, with the latter being responsible for the majority of oxidized adducts [25]. Oxidized mRNAs become abasic via the reaction described in Figure 1c whereby the peroxyl radical on the ribose is ejected, forming a carbocation that reacts with water to release the base. Levels of oxidative damage leading to abasic mRNA have also been correlated with environmental stress. For example, soybean plants exposed to cadmium have increased prevalence of mRNAs with abasic sites [26]. Exposure to heavy metals commonly increases production of reactive oxygen species; heavy metals stimulate the activity of NADPH oxidases [27] which generate superoxide anion and they deplete levels of reduced glutathione, a major antioxidant [28]. It has been postulated that there are three waves of reactive oxygen species production in response to heavy metal stress [29]. The first wave accumulates H_2_O_2_ through NADPH oxidase and the second accumulates superoxide radicals. The third wave involves the peroxidation of membranes from superoxide radicals leading to cell death. Chmielowska-Bak et al. [26] observed that the occurrence of oxidatively modified bases and abasic sites were separated in time such that high levels of oxidized guanosine (8-oxo-rG) were observed 3 h after cadmium application while increase in abasic sites was observed 24 h after heavy metal stress. These data correlate with the first and second wave of reactive oxygen species production and indicate sequential modification to mRNAs in response to stress [29].

mRNA can also be oxidized by cytochrome c-mediated peroxidation resulting in the loss of a guanosine residue to create an abasic site [30]. With cytochrome c as a catalyst in the presence of hydrogen peroxide, guanosine was the predominant ribonucleoside that was preferentially oxidized. 8-oxo-rG was subsequently depurinated leaving an abasic site. In addition to forming abasic sites in mRNAs, oxidized RNA can become covalently cross-linked to cytochrome c, resulting in its release from mitochondrial membranes. The dissociation of cytochrome c from the mitochondria into the cytosol triggers apoptosis [31]. Therefore, during oxidative damage, abasic mRNAs may initiate a protective signalling resulting in localized apoptosis or cell death.

Abasic mRNAs have also been observed in the R-loops of humans and yeast [32]. R-loops are three-stranded nucleic acid structures [33,34] which occur when the nascent RNA transcript anneals to its complementary single-stranded DNA template during transcription [35,36]. RNA-DNA hybrids are more stable than DNA-DNA hybrids [37] and can cause pausing and arrest of the RNA polymerase [35,38]. Human methylpurine DNA glycosylase (MPG) and apurinic/apyrimidinic endonuclease 1 (APE1) are components of the base excision repair pathway but are also associated with R-loops [32,39]. MPG has glycosylase activity on RNA of RNA – DNA hybrids, leading to RNA abasic sites whose ribose-phosphate backbones are subsequently cleaved by APE1 at the missing bases. Though the affinity of APE1 is higher for DNA than RNA, its activity on abasic RNAs resolves R-loops by initiating their degradation [32]. Recently, it has been shown that the abasic RNA sites in R-loops serve a regulatory role. Specifically, abasic sites increase the structural stability of R-loops which increases pausing of RNA polymerase II during transcription. For example, partial transcription of the non-coding enhancer RNA called AANCR of the gene apolipoprotein E (APOE) occurs upon N6-methyladenosine (m6A) modification which attracts MPG that subsequently cleaves the methylated base and generates an abasic site on the R-loop. The R-loops are resolved in response to stress conditions which stimulate full-length transcription of AANCR enhancer and hence, initiates APOE expression [40]. In addition to the AANCR, the transcription of more than 1000 other non-coding RNAs is regulated by abasic sites within RNA loops. Therefore, abasic RNAs can control expression of many genes by influencing the processivity of RNA polymerase II.

### Translation of abasic mRNAs

Following initiation of translation, protein synthesis relies on three repeating steps: 1) eEF1A, with GTP, binds an aminoacyl-tRNA to the ribosomal A site; 2) peptidyl transferase forms a peptide bond between the peptide at the ribosomal P site and the aminoacyl-tRNA at the A site, shifting the peptide to the A site; 3) eEF2, with GTP, translocates the peptidyl-tRNA with its mRNA codon to the P site leaving the A site empty for a new aminoacyl-tRNA. The processivity of translation involves the movement of mRNA through the ribosome and decoding of its sequence, one codon at a time (reviewed in [41]). Abasic sites in mRNA are therefore expected to affect aminoacyl-tRNA selection and binding of anticodon to codon sequences.

To investigate the impact of abasic sites on mRNA translation, a synthetic mRNA containing an abasic site in the middle of a coding sequence for five amino acids was tested in an *in vitro* translation assay [42]. Translation was completely inhibited by the abasic template. In addition to abasic sites, oxidized ribonucleotides were also tested and 5’-HO-rU, 5’HO-rC, 8-oxo-rA and 8-oxo-rG significantly inhibited but did not terminate translation. Therefore, abasic sites completely inhibit translation because they are non-coding lesions, whereas modifications of a base have a less severe effect on translation as they maintain hydrogen bonding patterns that allow a low level of ribosome elongation.

The effect of the position of a non-standard nucleotide within a codon was also investigated. Specifically, abasic nucleotides were introduced in the mRNA and investigated using mass spectrometry (MS) to identify the decoded amino acid [43]. When an abasic site was introduced in the third nucleotide position of the codon ‘A-U-A’ in the mRNA and translated using an *in vitro* bacterial system, 95% of the peptides were isoleucine (Ile) and only 5% were methionine (Met). By comparison, the eukaryotic HEK293T cells were unable to translate this modified codon, suggesting a different level of tolerance for abasic templates between prokaryotes and eukaryotes. Termination of translation has also been investigated by the insertion of abasic nucleotides at the stop codon [44]. Under normal conditions, release factor proteins recognize stop codons and bind to the ribosomal A site to facilitate cleavage between the nascent peptide and tRNA [45,46]. However, triple abasic sites at the stop codon were not recognized by either bacterial (RF1 and RF2) or eukaryotic-release factor (eRF1) and therefore peptides were not released from the stalled ribosomes [44]. Therefore, abasic sites in mRNAs inhibit both elongation and termination of translation.

Decrease in translation caused by modifications to mRNA has broader effects on gene expression. Specifically, accumulation of oxidized RNA, primarily 8-oxo-rG, correlated with a decrease in mRNA translatability which preferentially reduced levels of short-lived transcription factors. Some of these factors were repressors of transcription and their reduced levels resulted in enhanced transcription from genes controlled by these repressors [47]. Therefore, modifications to mRNA by loss of bases may also indirectly alter gene expression through more broad-ranging changes in transcription.

### Cellular detection of abasic mRNA during translation

**Figure 3.**
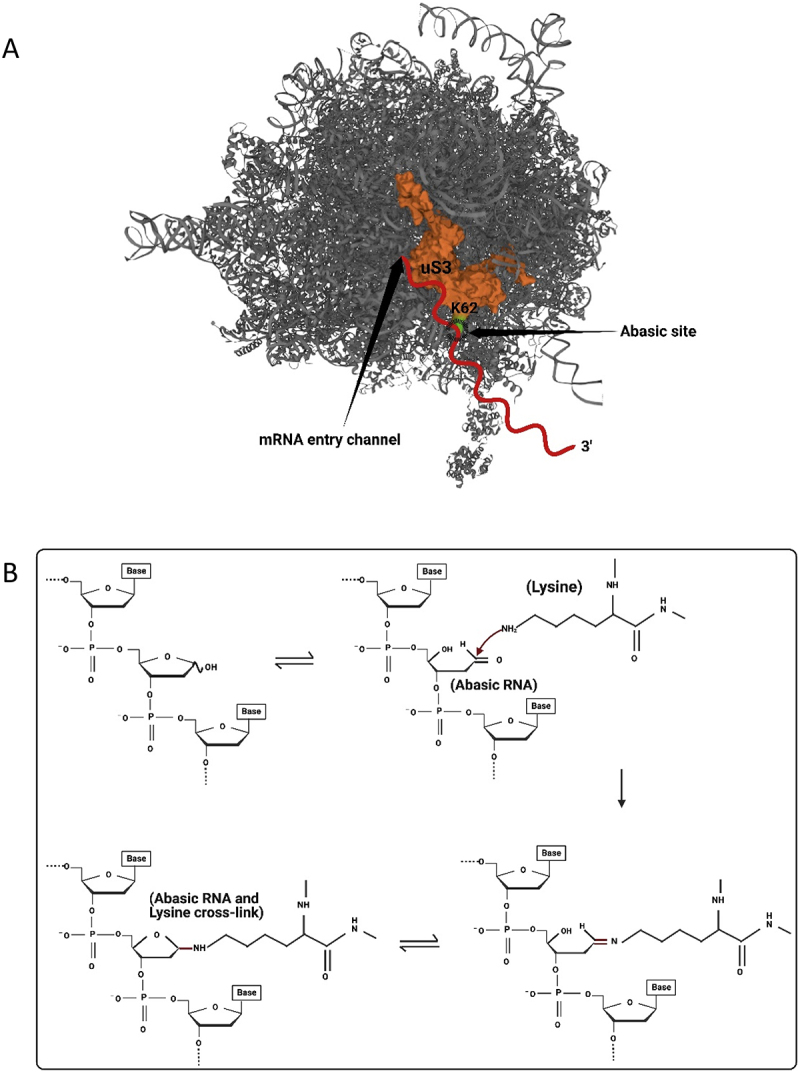
Abasic mRNA cross-linking with the ribosomal small subunit. A) an abasic nucleotide may cross-link with uS3, a small ribosomal subunit protein located near the mRNA entry site (PDB ID 4V6X). This cross-linking may stall the ribosome. mRNA is indicated as a red squiggly line and the site of cross-linking between Lysine 62 (shown in green) of uS3 and the abasic mRNA is circled. B) Detail of cross-linking between Lysine 62 of uS3 and the open conformation of an abasic ribose of the mRNA. Cross-linking involves a covalent Schiff base intermediate between the amino group of Lysine 62 and C1’ of the abasic site.

**Figure 4.**
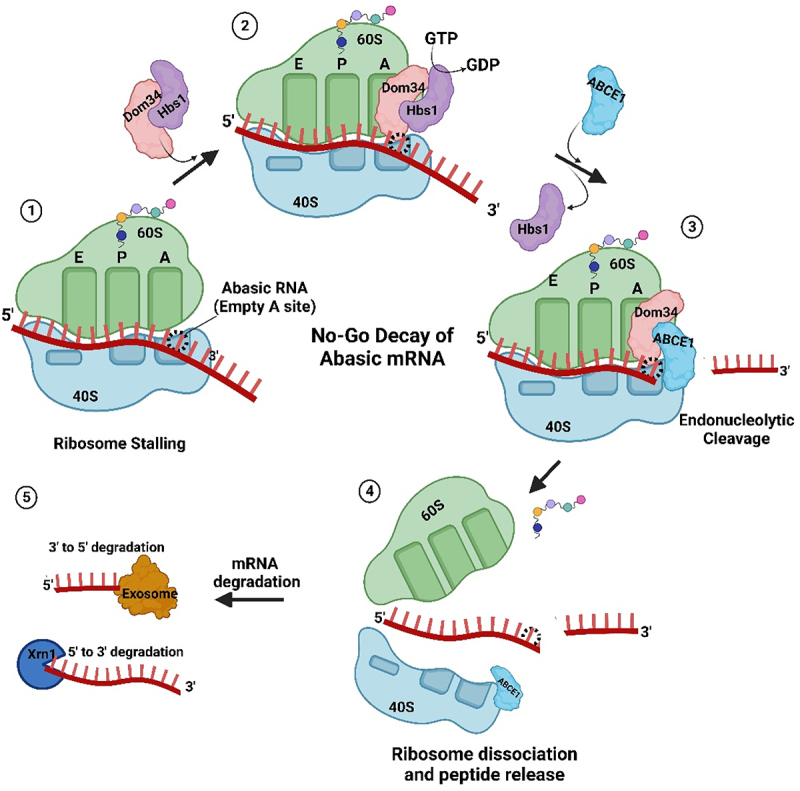
Abasic mRNA decay by the No-Go pathway. 1) Ribosome stalling with an abasic nucleotide at the A-site of the ribosome is recognized by Dom34p and Hbs1. 2) Hbs1 promotes binding of Dom34p to the A-site and 3) recruits ribosome-recycling factor ABCE1 for subsequent ribosome subunit dissociation. 4) Endonucleases, recruited through an unknown mechanism, cleave the mRNA 5) which is followed by exonuclease digestion from the 5’ and 3’ ends.

Recently, Ochkasova and colleagues [48,49] showed that abasic mRNA can cross-link to ribosomal protein uS3 (RpS3e), a component of the 40S ribosomal subunit (Figure 3a). uS3 is located at the mRNA entry site and stabilizes the preinitiation complex-mRNA contacts required for translation initiation [50]. Cross-linking between uS3 and abasic mRNA occurs when the abasic site is in the open aldehyde conformation and involves a covalent Schiff base intermediate between an amino group of rpS3 (Lysine 62) and C1’ of the abasic site followed by β-elimination of the 3’ phosphate (Figure 3b) [51]. Graifer and Karpova [52] suggested a model for abasic mRNA degradation during translation initiation: cross-linked ribosomal complexes are similar to stalled ribosomes and this phenomenon can induce No-Go decay which subsequently degrades the mRNA [53]. If the abasic site does not cross-link to the surface exposed region of uS3 at the mRNA entry tunnel, then it will likely be recognized at the A site of the ribosome. For example, enzymatically depurinated mRNA stalls elongating ribosomes at the abasic site, resulting in truncated protein synthesis and degradation of the mRNA via components of the No-Go decay pathway [54]. Stalling results in an empty A-site as no cognate aminoacyl-tRNA binds a codon with a missing base (Figure 4). This condition is recognized by Dom34p and Hbs1, the first complex of proteins shown to bind stalled ribosomes [55,56]. Dom34p bears structural similarity to eukaryotic-release factor 1 (eRF1), though it does not recognize stop codons [57,58]. Hbs1p is a G-protein with sequence similarity to translation release factor eRF3 [59,60]. GTP hydrolysis by Hbs1 promotes binding of Dom34p to the A site and recruits ribosome-recycling factor ABCE1 for subsequent ribosome subunit dissociation (Figure 4) [61,62]. Nucleases are recruited through an unknown mechanism. The recently identified endonuclease NONU−1, and its homolog Cue2 in yeast, may cleave upstream of stalled ribosomes at the abasic site and contribute to mRNA decay [63,64]. Therefore, abasic mRNA is recognized by the ribosome either during initiation or elongation and this surveillance prevents abasic mRNA translation.

## Abasic tRNA

Abasic sites are also found in some tRNAs as an intermediate step in their degradation. For example, yeast tRNA^Phe^ contains wybutosine, a hypermodified guanine residue at position 37 that affects translational fidelity by stabilizing the codon-anticodon base pairing in the decoding step of translation [65]. The modified base is susceptible to hydrolysis, which alters the tRNA structure [66]. tRNA^Phe^ is subsequently cleaved at the abasic site by aminoglycoside antibiotics [67]. Cleavage of the phosphodiester bond at the site of depurination formed from loss of the wybutosine base was 160-fold faster with physiological levels of neomycin B than by spontaneous cleavage [68]. Endonuclease cutting of tRNAs would prevent their function in translation. It is not known how aminoglycoside antibiotics cleave abasic tRNA; however, this activity would provide antibiotic producing cells with a competitive advantage against other cells.

Mature tRNAs that are not properly post-transcriptionally modified or contain sequence mutations are subject to rapid tRNA degradation (reviewed in [69]). In yeast, the degradation is mediated by methionine-requiring protein 22 (Met22) and two 5‘−3’ exonucleases, ribonucleic-acid-trafficking protein 1 (Rat1) and exonuclease 1 (Xrn1) [70]. Met22 is a phosphatase not directly involved in tRNA degradation; however, its activity on adenosine 3’,5’ bisphosphate (pAp) prevents accumulation of this metabolite which would inhibit Rat1 and Xrn1 function [71]. Another enzyme that facilitates tRNA degradation is the tRNA nucleotidyltransferase, which post-transcriptionally adds CCA to the 3’end of tRNAs. This modification is required for aminoacylation of tRNAs; however, the transferase can also add CCACCA to the 3’ends of unstable tRNAs. The resulting extended single-stranded end may facilitate degradation by exonucleases [72]. Given that modifications contribute to the thermostability of tRNAs, it is thought that unstable or non-functional tRNAs are detected largely by their altered structure [73]. It is not known whether abasic tRNAs would be detected and degraded in the same manner, though loss of a base is expected to cause significant alteration to structure given incomplete base pairing.

## Abasic rRNA

### Depurination of the α-sarcin/ricin loop of rRNA

Abasic rRNA is best exemplified by the activity of RNA N-glycosylases on the α-sarcin/ricin loop of the large 23S−28S rRNA. RNA N-glycosylases, often referred to as ribosome inactivating proteins, depurinate the rRNA at A4324 (mammalian rRNA numbering) of the large ribosomal subunit [74,75]. The α-sarcin/ricin loop is a component of the ribosomal GTPase associated centre [76] and removal of a base from this loop inhibits processivity of translation factors [14]. Several *in vitro* assays testing purified glycosylase activity in cell-free translation systems showed complete translation inhibition, which supported the idea that these enzymes were toxins synthesized to kill infected hosts or invading pathogens by inhibiting their translation [77,78].

Recent evidence suggests that cells detect damage to rRNA in advance of whole-scale translation inhibition. Specifically, expression of ricin in yeast resulted in only 10% depurination of rRNA which was sufficient to trigger cell cycle arrest [79]. Therefore, abasic rRNA signals to the cell, inhibiting yeast cell budding prior to complete translation inhibition. Though translation inhibition is irreversible as abasic rRNA does not seem to be repaired [80], a low level of damage with resulting cell stasis is reversible, as cells recover once ricin is no longer expressed, presumably through the synthesis of new ribosomes. Taken together, these results suggest that high level of abasic rRNA will kill cells due to translation inhibition whereas low levels will prompt signalling that alters the cell cycle.

### Ribotoxic stress response and downstream signalling

**Figure 5.**
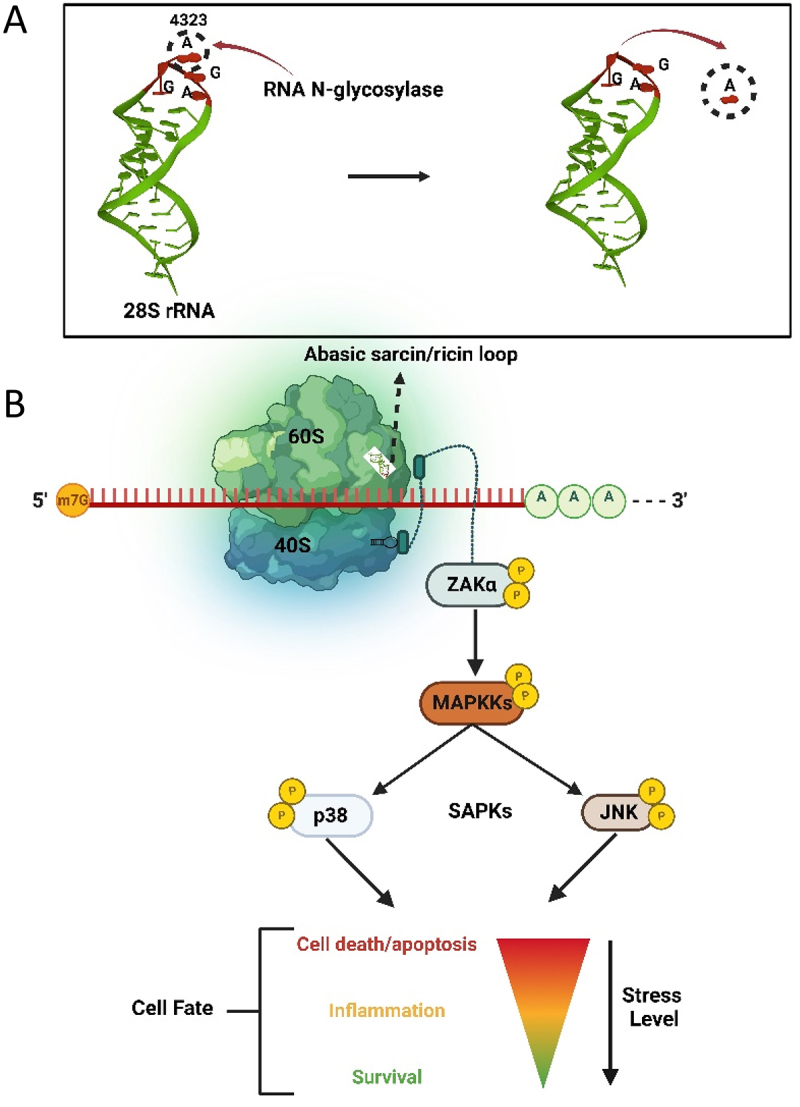
The ribotoxic stress response pathway. A) Hydrolysis of the adenine base (A4323) from the α-sarcin/ricin loop of 28S rRNA by an RNA N-glycosylase. B) Loss of a base from the α-sarcin/ricin loop (SRL) of the 28S rRNA is detected by the kinase ZAKα, which is bound to the ribosome. Through phosphorylation, damage to rRNA is signalled to downstream stress activated kinases JNK and p38, resulting in a proinflammatory response that may allow cell survival or terminate in apoptosis.

Since ribosomes play a central role in protein synthesis, conserved stress response signalling pathways have evolved to respond to obstructions to translation, which include detection of rRNA damage. Ribotoxic stress response (RSR) is a cellular signalling cascade in prokaryotes and eukaryotes that is activated following loss of a base or structural damage to the α-sarcin/ricin loop (SRL) of the 28S rRNA [81] (Figure 5). The MAP kinase sterile-motif alpha and leucine zipper kinase (ZAK) is activated by ribotoxic stress and initiates a downstream signalling response from defective ribosomes [82]. ZAK has two splice variants, ZAKα and ZAKβ, which differ in length of their C-termini [83]. ZAKα binds directly to the ribosome via a cryptic C-terminal RNA binding domain [84]. A mutant ZAKα lacking its C-terminal extension did not bind ribosomes nor elicit downstream signalling, indicating that ZAKα is the sensor of ribosome damage [84]. Jandhyala et al. [85] demonstrated that chemical inhibition and small interfering RNA (siRNA) knockdown of ZAK prevented activation of downstream kinases in response to ribotoxins ricin and Stx. Furthermore, zak-/- mice did not experience activation of stress-activated protein kinases c-Jun NH2-terminal kinases (JNKs) and p38 following ricin exposure, supporting the involvement of ZAK in signalling RSR. Abasic rRNA leads to activation of a downstream MAPK signalling cascade that initially triggers a proinflammatory response and strongly activates JNK and p38 [86,87]. The extent of damage to ribosomes determines the cell outcome. That is, limited damage results in temporary cell stasis and the opportunity for cells to recover. For example, microbial ribotoxins that halt translation elongation activate ZAKα which hyperphosphorylates NLRP1, a nucleotide-binding domain and leucine-rich repeat protein that is a key mediator of innate immunity [88]. Therefore, a link exists between RSR and antimicrobial defence mediated through inflammation. However, prolonged damage to the α-sarcin/ricin loop, as would occur with continued ricin exposure, elevated production of proinflammatory cytokines and led to apoptotic cell death [82,85].

## Concluding remarks

mRNAs, tRNAs and rRNA may become abasic during their lifetime and though this damage can be a consequence of normal metabolism, their levels increase during periods of cell stress. Cells have ways to detect this damage and even though RNAs do not seem to be repaired like DNA, the time between loss of a base and degradation of the RNA is enough to elicit signalling pathways in cells. Specifically, we have seen how abasic mRNAs may cross-link to ribosomal proteins or cause ribosomes to stall when they encounter missing bases at their A site. In addition, abasic mRNAs within R-loops can pause the RNA polymerase and determine which messages get transcribed. Though abasic mRNAs reduce levels of their individual protein products, damage to rRNA in the form of α-sarcin/ricin loop depurination will have wider impact on cellular translation. Known as the ribotoxic stress response, rRNA damage triggers a kinase cascade resulting in inflammation and potentially cell death. Though we have some understanding of how abasic RNAs are detected, we are just beginning to ask how this damage is communicated within cells and what controls the balance between cell recovery and adaptation versus apoptosis. Several interesting questions remain to be answered.

1. Recent results show that the MAPKKK ZAKα signals rRNA depurination through autophosphorylation [84], but how ZAKα detects specific structural change to rRNA resulting from damage remains unknown. The balance between downstream stress signalling that leads to cell survival versus apoptosis is also not clearly defined. In addition, it is assumed that recovery of cells from exposure to RNA N-glycosylases, such as ricin, involves turnover of ribosomes. It is uncertain how much depurination of rRNA is tolerated by cells and when synthesis of new ribosomes initiates to compensate for damaged 28S rRNA.
2. How abasic mRNAs are detected and degraded needs further investigation. Though No-Go decay pathway appears integral to the process and may involve collision of ribosomes upstream of the ribosome at the abasic site, the endonucleases that cleave abasic mRNAs remain partially identified. Specifically, the yeast endonuclease Cue2 was recently shown to cleave mRNA at the ribosome upstream of the stalled ribosome [64]. However, deletion of Cue2 did not stabilize mRNA within a stalled ribosome, suggesting the existence of other yet unidentified nucleases. Alternatively, perhaps endonucleolytic cleavage of abasic mRNA is not required for disassembly of ribosomes stalled at an abasic site. In addition, it is not known whether abasic mRNAs are detected only during their translation. Recent evidence describes how oxidized mRNAs are recognized by RNA-binding proteins which are either involved in degradation of the oxidized mRNA or initiating apoptosis depending on the degree of mRNA modification [89,90]. Whether abasic mRNAs are similarly sequestered remains to be investigated.
3. The discovery of abasic RNA within R-loops that form during transcription is the first to show that this RNA modification can affect the elongation of RNA polymerase II [40]. Abasic sites stabilize R-loops causing the RNA polymerase to pause and prevent the synthesis of full-length transcript. A change in environmental condition, such as the application of hyperosmotic stress, resolves these R-loops and allows downstream transcription of genes. Given that this mechanism of regulation over transcription elongation is different from the well-characterized pausing of RNA polymerase at promoters, questions remain about its mechanism. For example, how does change in stress level resolve R-loops? In addition, it is currently unclear whether the tendency of some nascent transcripts to form abasic sites is characteristic of their specific modifications, such as methylation.

Continued research will provide answers to these and other questions about how abasic RNAs are detected, how they function to communicate cell status and control gene expression before they are eventually degraded. However, it is clear that abasic RNA levels change during cell stress and given the chemical stability of this modification, abasic RNAs may act as signalling molecules that allow cells to either adapt or succumb to environmental change.

## Data Availability

Data sharing is not applicable to this article as no new data were created or analysed in this study.
